# Classifying genes to the correct Gene Ontology Slim term in *Saccharomyces cerevisiae *using neighbouring genes with classification learning

**DOI:** 10.1186/1471-2164-11-340

**Published:** 2010-05-28

**Authors:** Heather A Amthauer, Costas Tsatsoulis

**Affiliations:** 1 Department of Computer Science, Frostburg State University, Frostburg, Maryland, USA; 2 Department of Computer Science and Engineering, University of North Texas, Denton, Texas, USA

## Abstract

**Background:**

There is increasing evidence that gene location and surrounding genes influence the functionality of genes in the eukaryotic genome. Knowing the Gene Ontology Slim terms associated with a gene gives us insight into a gene's functionality by informing us how its gene product behaves in a cellular context using three different ontologies: molecular function, biological process, and cellular component. In this study, we analyzed if we could classify a gene in *Saccharomyces cerevisiae *to its correct Gene Ontology Slim term using information about its location in the genome and information from its nearest-neighbouring genes using classification learning.

**Results:**

We performed experiments to establish that the MultiBoostAB algorithm using the J48 classifier could correctly classify Gene Ontology Slim terms of a gene given information regarding the gene's location and information from its nearest-neighbouring genes for training. Different neighbourhood sizes were examined to determine how many nearest neighbours should be included around each gene to provide better classification rules. Our results show that by just incorporating neighbour information from each gene's two-nearest neighbours, the percentage of correctly classified genes to their correct Gene Ontology Slim term for each ontology reaches over 80% with high accuracy (reflected in F-measures over 0.80) of the classification rules produced.

**Conclusions:**

We confirmed that in classifying genes to their correct Gene Ontology Slim term, the inclusion of neighbour information from those genes is beneficial. Knowing the location of a gene and the Gene Ontology Slim information from neighbouring genes gives us insight into that gene's functionality. This benefit is seen by just including information from a gene's two-nearest neighbouring genes.

## Background

Determining novel gene functionality is critical for bringing a better understanding of how an organism functions as a whole. Traditional biological approaches to determining gene functions mainly focus on testing specific hypotheses through well designed mutagenesis experiments. However, methods of this kind suffer from the high cost of labour and funds. With the proliferation of protein and nucleic acid sequences catalogued in genome databases, the investigation of the function of a gene and its encoded product often begins by comparing its sequence with those of previously characterized genes. But, the search for homologues does not always reveal information about function. As noted by Alberts *et al*. [1] in the *Saccharomyces cerevisiae *genome, "30% of the previously uncharacterized genes could be assigned a putative function by homology analysis; 10% had homologues whose function was also unknown; and another 30% had no homologues in any existing databases (the remaining 30% of the genes had been identified before sequencing the yeast genome)." Sequence similarity alone cannot provide full function specificity [2]. The predictions that emerge from sequence analysis are often only a tool to direct further experimental investigations.

Knowing the Gene Ontology Slim terms associated with a gene give us insight into how its gene product behaves in a cellular context using three different ontologies: molecular function, biological process, and cellular component. These terms describe where a gene product is located or its association with cellular components, they describe its activity in biological processes and the molecular functions it performs during the biological processes. The Gene Ontology Slim give an "overview of the ontology content" and are useful for summarizing the results of Gene Ontology annotation [3]. In the context of this paper the term function is used to refer to all aspects and concepts described by the Gene Ontology classifications. The Gene Ontology comprises a set of well-defined terms with well-defined relationships. The structure of Gene Ontology reflects the current representation of biological knowledge as well as serving as a guide for organizing new data [4]. The vocabulary is fluid and undergoes consistent revision. The intention of the Gene Ontology is to make possible, in a flexible and dynamic way, the annotation of homologous gene and protein sequences in multiple organisms using a common vocabulary. It has become a broadly accepted classification system for function assignment.

Several studies have noted that gene location in higher eukaryotic organisms is not random and suggest that there may be patterns in gene location [5-14]. Several research groups have further noted that genes with related function are often located close to each other on the chromosomes [15-19]. Also functional overlaps (shared Gene Ontology terms) have been found between clustered genes in yeast when examining spans of small chromosomal distances (less than 10 kbp) [20]. These studies suggest that the location of a gene and its surrounding neighbourhood of genes have an influence on its functionality, but none of these studies determined if there are true patterns within a genome, and if we can learn from these patterns. If there are patterns based on how genes cluster/group within a genome, then we can generate rules and relationships based on these patterns through classification learning.

In this study, we analyzed if we could classify a gene in *Saccharomyces cerevisiae *to its correct Gene Ontology term using information about its location in the genome and information from its nearest-neighbouring genes using MultiBoositng with C4.5. This methodology can assist researchers by expediting the process of determining the functionality of a gene by providing classification rules that will determine a gene's Gene Ontology term.

## Methods

### Classification Techniques

In classification learning, the learning system is presented with a set of classified examples. From these examples, the system is expected to learn a way of classifying unseen examples [21]. We used a popular classification learning technique that combines MultiBoosting [22] with the decision tree classifier C4.5 [23]. MultiBoosting combines AdaBoost (a boosting technique) [24] with wagging (a variant of bagging) [25] to form decision committees. This technique "boosts" a learning algorithm to a stronger learning algorithm by taking advantage of AdaBoost's high bias and variance reduction and wagging's strong variance reduction. It has been shown that when using the C4.5 algorithm as its base learning algorithm, MultiBoosting produces superior decision committees [22].

In building decision trees, C4.5 determines which attribute to split on given a set of examples with different classes. This attribute is selected based on information measured in bits [26]. For each attribute, the C4.5 algorithm calculates the information gain from splitting the tree on that attribute. The best attribute has the highest information gain. A decision node is created that splits the dataset on that best attribute. The process is repeated on the sub-trees of that node.

The versions of these algorithms that we used can be found in WEKA (the Waikato Environment for Knowledge Analysis). WEKA is open source software issued under the GNU General Public License. It is a collection of machine learning algorithms for data mining tasks. WEKA contains tools for data pre-processing, classification, regression, clustering, association rules, and visualization [21]. The WEKA version of MultiBoosting is MultiBoostAB and the WEKA version of C4.5 is called J48.

### Classification experiments

Experiments were performed to establish if MultiBoostAB using the J48 classifier could correctly classify Gene Ontology Slim terms of a gene. Different neighbourhood sizes were examined to determine how many nearest neighbours should be included around each gene to provide better classification rules. As a baseline, the classification process was performed using no neighbour information. Also, the classification process was performed using the entire genome, and then it was repeated on each chromosome to examine if partitioning the classification process would yield better rules.

The classifier was trained using different neighbourhood attributes. A gene's neighbourhood attributes were determined by the attributes of its nearest neighbours on both strands and both upstream and downstream to the gene. The gene's attributes that were used in the training process were: its chromosome number (one, two, three, four, five, six, seven, eight, nine, ten, eleven, twelve, thirteen, fourteen, fifteen, sixteen, seventeen (seventeen is the mitochondrial chromosome)), its start position (in bp), its stop position (in bp) and its strand ("W" for Watson and "C" for Crick), the gene's Gene Ontology aspect (ontology: cellular component (C), biological process (P), or molecular function (F)) and the gene's Gene Ontology Slim term. The neighbour attributes that were used for the training process were: the neighbour's number (1-10, with 1 representing the closest neighbour), the neighbour's strand, the neighbour's distance (in bp) from the gene (determined from the mid-point of each), the neighbour's Gene Ontology aspect, the neighbour's Gene Ontology Slim term.

The size of the neighbourhood was determined by how many nearest neighbours should be included. All experiments were repeated using different sizes of neighbourhoods. These neighbourhoods included information from the nearest neighbours, the two-nearest neighbours, the five-nearest neighbours on both strands and both upstream and downstream from the gene, and when analyzing the individual chromosomes, the ten-nearest neighbours of each gene were also included.

The parameters for the MultiBoostAB algorithm using the J48 classifier were set to use reweighting instead of resampling because past experiments produced results that suggested "reweighting is more effective than resampling" [22]. The other settings were set to default values.

### Datasets

We used publicly available data pertaining to gene location and Gene Ontology Slim terms available at the *Saccharomyces *Genome Database http://www.yeastgenome.org/. The *Saccharomyces cerevisiae *genome was selected for classification because of all the sequenced genomes, it has the most ideal characteristics for a test case. The *Saccharomyces cerevisiae *genome shows a high amount of clustering of genes that are involved in the same metabolic pathway [9], and it shows clustering of essential genes into regions of low recombination [27]. Incidences of highly coordinated expression of linked genes have also been found in yeast [8,28]. The files (SGD_features.tab and go_slim_mapping.tab) were obtained from the Anonymous FTP site [29]. Information was extracted from the files and formatted as comma-separated values (CSV) files to be compatible with WEKA. A sample of the file format can be seen in Table 1.

**Table 1:** File format information.

Chromosome Number	Start	Stop	Strand	Neighbour number	Neighbour strand	Distance	Neighbour GO aspect	Neighbour GO Slim term	GO aspect	GO Slim term
ten	18536	16767	C	1	W	2697	C	plasma membrane	C	cellular component

ten	18536	16767	C	1	W	2697	C	plasma membrane	F	hydrolase activity

ten	18536	16767	C	1	W	2697	C	plasma membrane	P	biological process

ten	18536	16767	C	1	W	2697	F	transporter activity	C	cellular component

ten	18536	16767	C	1	W	2697	F	transporter activity	F	hydrolase activity

ten	18536	16767	C	1	W	2697	F	transporter activity	P	biological process

ten	18536	16767	C	1	W	2697	P	transport	C	cellular component

ten	18536	16767	C	1	W	2697	P	transport	F	hydrolase activity

ten	18536	16767	C	1	W	2697	P	transport	P	biological process

ten	18536	16767	C	2	W	2697	?	?	C	cellular component

ten	18536	16767	C	2	W	2697	?	?	F	hydrolase activity

ten	18536	16767	C	2	W	2697	?	?	P	biological process

Shows the format of the data files that included neighbour's Gene Ontology information and gene's Gene Ontology information. The columns represent the different values that were separated by commas. "?" represent unknown values.

### Metrics

The performance of the classification process can be evaluated by two metrics: percentage of correctly classified instances and the F-measure. The percentage of correctly classified instances is a basic accuracy measurement that can be determined by the following:

The F-measure is a weighted harmonic mean of precision and recall. It is calculated in the following manner:

Where precision and recall are calculated in the following manner

In statistics, the F-measure is a measure of a test's accuracy, where an F-measure reaches its best value at 1.0 and worst score at 0.

## Results and Discussion

### Classifying Genes to Correct Gene Ontology Terms

The results of the experiments are based on averages of ten different runs of each dataset being randomized and then split for training and testing. The size of the datasets varied depending on what experiments were being performed. The dataset that contained no neighbour information contained 31554 instances. This number increased as more neighbour information was included. For instances where there was missing attribute values, the missing values were substituted with a "?". The training sets were set to contain 66% of the instances randomly selected from each dataset. In this analysis, the classification of Gene Ontology Slim terms for all three ontologies are combined. The MultiBoostingAB algorithm using the J48 classifier generated different trees for each data set it analyzed. The trees generated for the individual chromosomes did share similar structures in that the root node was the Gene Ontology Slim term for the given gene followed by start positions that partitioned the chromosome and then the neighbouring gene's Gene Ontology Slim term.

**Figure 1:**
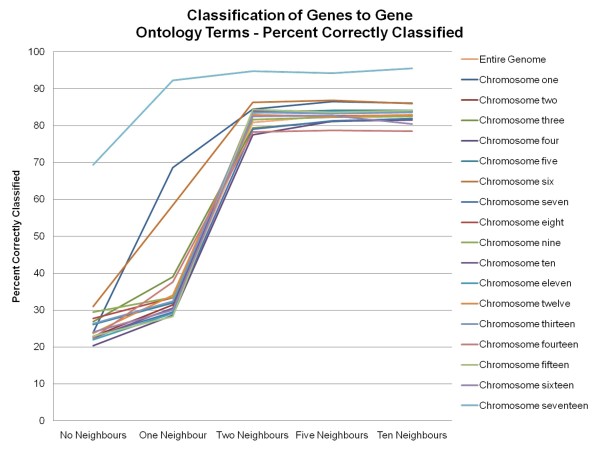
**Percent of Correctly Classified Genes to Gene Ontology Slim Terms**. Percent of correctly classified genes to Gene Ontology Slim terms for the entire genome and the individual chromosomes (chromosome seventeen represents the mitochondrial chromosome) using the MuliBoostAB algorithm using the J48 classifier.

**Figure 2:**
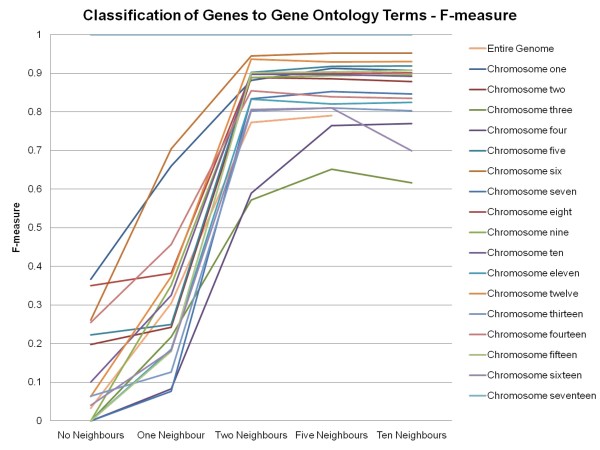
**F-measures of Classified Genes to Gene Ontology Slim Terms**. F-measures of classified genes to Gene Ontology Slim terms for the entire genome and the individual chromosomes (chromosome seventeen represents the mitochondrial chromosome) using the MuliBoostAB algorithm using the J48 classifier.

The results of this study also showed that in classifying genes to their correct Gene Ontology Slim term, the inclusion of neighbour information from those genes is beneficial (See Figure 1). By incorporating neighbour information from each gene's two-nearest neighbours, the percentage of correctly classified genes increases to over 80% for most chromosomes. A gene is considered to be correctly classified if all of its Gene Ontology Slim terms have been predicted. This phenomenon of having the incorporation of neighbour information being beneficial is also seen in the F-measures obtained by the classifier (See Figure 2). Partitioning of the classification process by chromosome produces better accuracy results than using the classification results generated when using information from the entire genome. By incorporating neighbour information from each gene's two-nearest neighbours, the F-measures increase to over 0.80 for most chromosomes.

The inclusion of a neighbour's Gene Ontology information being beneficial in classifying genes to the correct Gene Ontology Slim term supports finding from other studies. In a study by Fukuoka *et al*., they investigated Gene Ontology categories of gene pairs that were considered highly correlated in chromosomal distance ranges between 0 and 20 kbp and between 980 and 1000 kbp. The results of the pairwise analysis of Gene Ontology category showed that only highly correlated pairs shared the same category and most of these pairs were not duplicates, meaning the genes did not share a common history; this was determined by BLAST analysis [20].

### Accuracy of the Classification of Genes to Specific Gene Ontology Slim terms

**Figure 3:**
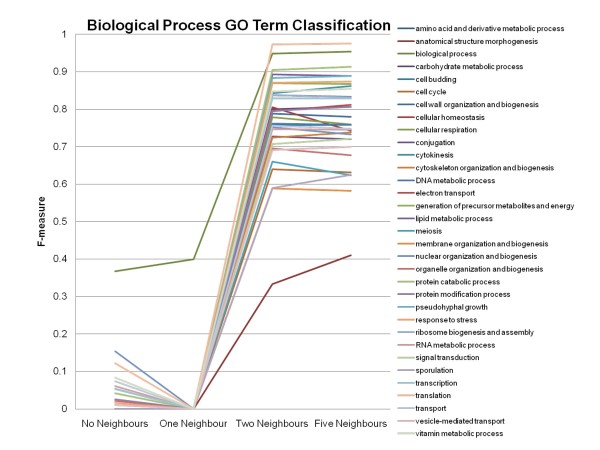
**Accuracy of the Classification of Genes to Biological Process Gene Ontology Slim Terms**. F-measures obtained using the MultiBoostAB algorithm using the J48 classifier for the individual Gene Ontology (GO) Slim terms.

**Figure 4:**
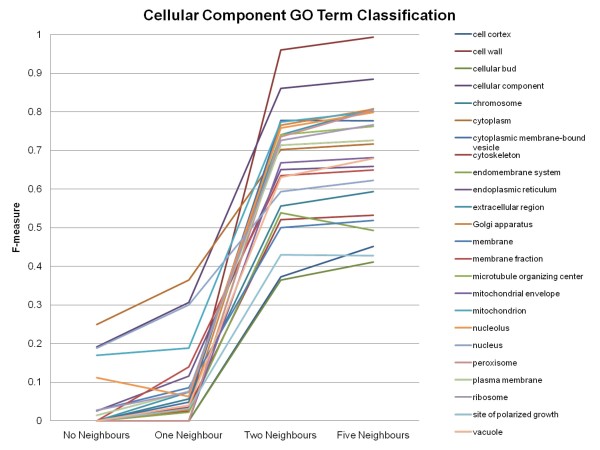
**Accuracy of the Classification of Genes to Cellular Component Gene Ontology Slim Terms Continued**. F-measures obtained using the MultiBoostAB algorithm using the J48 classifier for the individual Gene Ontology (GO) Slim terms continued.

**Figure 5:**
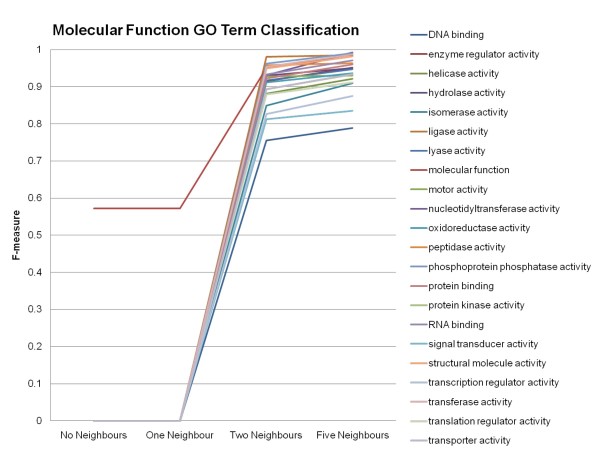
**Accuracy of the Classification of Genes to Molecular Function Gene Ontology Terms Continued**. F-measures obtained using the MultiBoostAB algorithm using the J48 classifier for the individual Gene Ontology (GO) Slim terms continued.

To see the effect on the accuracy of classification for each Gene Ontology Slim term using information from the entire genome, the F-measures obtained by the MultiBoostAB algorithm using the J48 classifier for each Gene Ontology Slim term can be examined. Most of the Gene Ontology Slim terms benefited from the addition of neighbour information (See Figures 3, 4 and 5). Based on the results, the inclusion of neighbour information beyond each feature's two-nearest neighbours did not drastically increase the accuracy of classification of genes to their correct Gene Ontology Slim terms. When no neighbour information is included, Gene Ontology Slim terms that represent broader functionalities, biological process, molecular function, have higher accuracy scores compared to that of Gene Ontology Slim term of cellular component, meaning the classifier was able to classify genes that belonged to these broader Gene Ontology Slim terms better. The four Gene Ontology Slim term groups that obtained the lowest F-measures (did not achieve an F-measure above 0.5) are: anatomical structure morphogenesis, cell cortex, cellular bud and site of polarized growth, meaning the inclusion of neighbour information did not improve the classification process for genes belonging to these Gene Ontology terms as much as it did for the genes belonging to other terms.

### Classification of Genes to Gene Ontology Slim Terms Removing Gene Location

**Figure 6:**
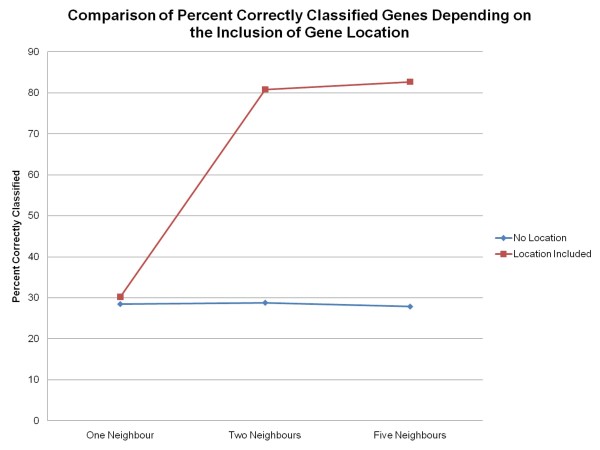
**Percent of Correctly Classified Genes to Gene Ontology Slim Terms**. Percent of correctly classified genes to Gene Ontology Slim terms for the entire genome with and without the inclusion of the gene's location using the MuliBoostAB algorithm using the J48 classifier.

**Figure 7:**
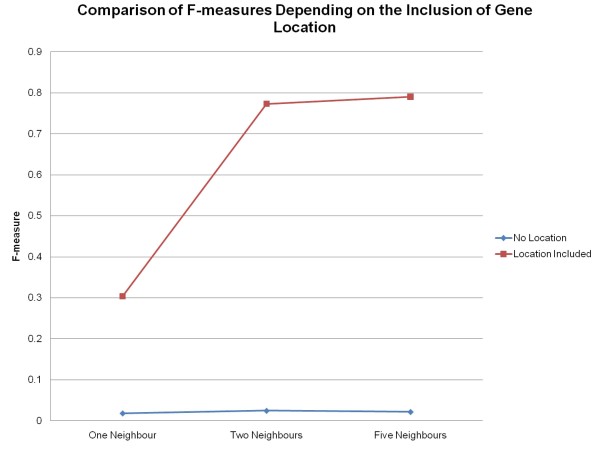
**F-measures of Classified Genes to Gene Ontology Slim Terms**. F-measures of classified genes to Gene Ontology Slim terms for the entire genome with and without the inclusion of the gene's location using the MuliBoostAB algorithm using the J48 classifier.

A series of experiments was performed to determine if a gene could be classified to its Gene Ontology Slim term when given only neighbour information for training. The attributes that were used for the training process were: the neighbour's number, the neighbour's strand, the neighbour's distance (in bp) from the gene, the neighbour's Gene Ontology aspect, the neighbour's Gene Ontology Slim term, the gene's Gene Ontology aspect and the gene's Gene Ontology Slim term. Without the gene's location for training, the percentage of correctly classified genes to their correct Gene Ontology Slim term is reduced to below 30% (See Figure 6). The accuracy of the classification is poor, obtaining F-measures below 0.025 (See Figure 7).

## Conclusions

In this study, the effect of information from neighbouring genes influencing a gene's Gene Ontology Slim terms was examined using classification learning. We confirmed that knowing the Gene Ontology Slim information from a gene's surrounding genes allows the MultiBoostAB algorithm using the J48 classifier to correctly classify a gene's Gene Ontology Slim term over 80% of the time. These classification results are obtained by just including Gene Ontology Slim information from each gene's two-nearest neighbours. This study demonstrates that there are true patterns within the yeast genome. We can generate rules based on these patterns through classification learning that can provide us with more insight to how genes cluster within a genome. Since other genomes (*e.g*. *Homo sapiens*, *Caenorhabditis elegans*, *Drosophila melanogaster*, *Arabidopsis thaliana*) exhibit clustering patterns [5-19], this methodology should translate to those other genomes that are annotated. The optimal neighbourhood size incorporated for each genome would have to be established through experimentation.

## Authors' contributions

HA conceived the methodology, ran and analyzed the results of the classification experiments and drafted the manuscript. CT provided machine learning expertise and guided the study. All authors read and approved the final manuscript.
